# Electrophoretic deposition of carbon nanotube on reticulated vitreous carbon for hexavalent chromium removal in a biocathode microbial fuel cell

**DOI:** 10.1098/rsos.170798

**Published:** 2017-10-25

**Authors:** Kangqing Fei, Tian-shun Song, Haoqi Wang, Dalu Zhang, Ran Tao, Jingjing Xie

**Affiliations:** 1 State Key Laboratory of Materials-Oriented Chemical Engineering, Nanjing Tech University, Nanjing 211816, People's Republic of China; 2 College of Life Science and Pharmaceutical Engineering, Nanjing Tech University, Nanjing 211816, People's Republic of China; 3 Key Laboratory of Bio-based Materials, Qingdao Institute of Bioenergy and Bioprocess Technology, Chinese Academy of Sciences, Qingdao 266101, People's Republic of China; 4 International Cooperation Division, China National Center for Biotechnology Development, Beijing 100039, People's Republic of China; 5 Nanjing Foreign Language School, Nanjing 210018, People's Republic of China; 6 Jiangsu National Synergetic Innovation Center for Advanced Materials (SICAM), Nanjing 211816, People's Republic of China

**Keywords:** microbial fuel cell, Cr(VI) removal, carbon nanotube, reticulated vitreous carbon, electrophoretic deposition

## Abstract

For Cr(VI)-removal microbial fuel cell (MFC), a more efficient biocathode in MFCs is required to improve the Cr(VI) removal and electricity generation. RVC-CNT electrode was prepared through the electrophoretic deposition of carbon nanotube (CNT) on reticulated vitreous carbon (RVC). The power density of MFC with an RVC-CNT electrode increased to 132.1 ± 2.8 mW m^−2^, and 80.9% removal of Cr(VI) was achieved within 48 h; compared to only 44.5% removal of Cr(VI) in unmodified RVC. Cyclic voltammetry, energy-dispersive spectrometry and X-ray photoelectron spectrometry showed that the RVC-CNT electrode enhanced the electrical conductivity and the electron transfer rate; and provided more reaction sites for Cr(VI) reduction. This approach provides process simplicity and a thickness control method for fabricating three-dimensional biocathodes to improve the performance of MFCs for Cr(VI) removal.

## 1. Introduction

Hexavalent chromium (Cr(VI)), which is a well-known carcinogen and mutagen that threatens public health, has high water solubility and mobility in the environment [1]. The common method for Cr(VI) removal is Cr(VI) reduction to the less toxic trivalent chromium (Cr(III)) by electrochemical [2] or biological reduction [3]. However, the electrochemical reduction method requires an acidic solution for treatment and needs energy input, whereas the biological reduction method needs an efficient Cr(VI)-reducing bacterial species through long acclimation. These shortcomings have promoted researchers to look for other alternatives.

Recently, an alternative method for reducing Cr(VI) using microbial fuel cells (MFCs) [4–6] with abiotic cathode has been proposed, in which Cr(VI) is fed to its cathode and functions as the electron acceptor due to the high redox potential of the Cr(VI)/Cr(III) couple. At natural pH, the Cr(VI) reduction rate can be further enhanced by a biocathode [7–10], where bacteria as biocatalysts can accelerate the reduction in Cr(VI) to Cr(III). The biocathode MFC eliminates the input of energy to and pH adjustment in the acid condition and offers the advantages of electricity generation, low operating cost and self-regenerating biocatalyst. However, biocathode MFC is still in its infancy. Thus, extensive research is required to find a more efficient biocathode in MFCs for improving Cr(VI) removal and electricity generation.

The characteristics of a biocathode surface affect its bacterial attachments, biofilm development and the interaction between a microorganism and an electrode [11]. Owing to its good biocompatibility and corrosion-resistance, carbon-based materials are the most commonly selected electrodes for Cr(VI)-removal MFC. Typically, graphite felt, graphite fibre, plate and granules [7,12–14] have been used as biocathode materials. Nevertheless, electrode materials and their effects on the performance of Cr(VI)-removal MFC can still be enhanced.

Reticulated vitreous carbon (RVC) is a rather cheap and commercially available open-pore foam carbon material that is used in MFCs [15–18]. It has several advantages for MFC, such as a very high surface area to volume ratio and strong chemical and heat resistance. However, due to its relatively low electrical conductivity and low surface roughness, RVCs require further surface modification for electron transfer and microbial attachment. Nanostructured materials, such as graphene [19–21] and carbon nanotubes (CNTs) [22–24], have promising properties as catalysts and enhance the electrode activity in MFC applications. CNTs have higher active surface areas, excellent conductivity and are highly biocompatible, which allow for bacteria immobilization and proliferation. The most common method for CNT electrode modification is electrophoretic deposition (EPD) [25–28], which constructs the highly uniform deposited CNT layers. Compared to other processing methods for CNTs, EPD is relatively easy to perform and has simple equipment requirements. Furthermore, it is capable of fabricating three-dimensional CNT-RVCs which may benefit the Cr(VI) reduction in MFC.

To demonstrate this concept, CNT-modified RVC electrodes were prepared by EPD and further used as biocathodes in MFC for Cr(VI) removal. The electricity generation and Cr(VI) removal rate in the MFC with the CNT-modified RVC electrode were examined and compared to those obtained in the MFC with an unmodified RVC cathode. In addition, the influences of the different electrode materials were discussed through an in-depth analysis.

## 2. Material and methods

### 2.1. MFC construction and operation

The structure of the dual-chamber MFC is similar to that reported by Song *et al*. [10]. The dual-chamber MFC was made of plexiglas material (net volume of 120 ml each). The two chambers were separated by a proton exchange membrane (Nafion117, Dupont Co., USA). The anode was made of graphite felt (40 mm × 40 mm × 5 mm, length × width × thickness). An RVC-CNT biocathode or RVC biocathode (50 mm × 25 mm × 10 mm, length × width × thickness) was placed in the cathode chamber. The anode chamber of the MFC was inoculated with 5 ml of anaerobic activated sludge and 115 ml of glucose culture medium (pH 7.0, per litre of deionized H_2_O) consisting of 0.31 g NH_4_Cl, 11.53 g Na_2_HPO_4_·12H_2_O, 2.77 g NaH_2_PO_4_·2H_2_O, 0.13 g KCl and 1 g glucose. The cathode medium (11.53 g l^−1^ Na_2_HPO_4_·12H_2_O, 0.28 g l^−1^ NH_4_Cl, 2.77 g l^−1^ NaH_2_PO_4_·2H_2_O, 0.39 g l^−1^ KCl, 0.1 g l^−1^ NaHCO_3_) containing 20 mg l^−1^ Cr(VI) (prepared by dissolving K_2_Cr_2_O_7_ in deionized water) was added to the cathode chamber. The MFCs were operated at a fixed external resistance of 1000 Ω and maintained at 30°C. All experiments were carried out in duplicate under each experimental condition.

### 2.2. Preparation of RVC-CNT biocathode

The RVC-CNT electrode was manufactured by EPD technique. The RVC was activated by immersion in 2 M HNO_3_ for 8 h, and washed with deionized water. Then, the RVC was collected and oven-dried. Carboxylation multi-walled carbon nanotubes (MWCNTs) were purchased from Nanjing XFNANO Materials Tech Co., Ltd. The MWCNTs were ultrasonically dispersed in deionized water at a concentration of 100 mg l^−1^ for 1 h at 25°C. The RVC was used as the anode and the stainless-steel mesh was used as the cathode. They were immersed into the MWCNT suspension simultaneously. The distance between the two electrodes was 10 mm. A DC power supply of 30 V was applied to the two electrodes for 10 min. The MWCNTs began to deposit on the RVC electrode. Then, RVC-CNT electrode was taken from the suspension and dried in vacuum.

After EPD, an *ex situ* acclimation method according to our previous report [8] was used to obtain Cr(VI)-removal biocathodes. The RVC-CNT electrode was used as anode in the dual-chamber MFC, and inoculated with 5 ml anaerobic activated sludge and 115 ml glucose culture medium for acclimatization. The cathode was graphite felt and filled with 40 mM ferricyanide medium in the cathode chamber (2.452 g l^−1^ NaH_2_PO_4_·12H_2_O, 4.576 g l^−1^ Na_2_HPO_4_·2H_2_O, 0.13 g l^−1^ KCl, pH 7.0). The other conditions were the same as those above. After the MFC achieved the steady state for power generation, the RVC-CNT electrode was then removed from the anode chamber and gently cleaned with deionized water. After cleaning, this RVC-CNT electrode was transferred to the cathode chamber and used as the RVC-CNT biocathode in the Cr(VI)-removal MFC. For control, the acclimation method of RVC biocathode used was the same as that of the RVC-CNT biocathode.

### 2.3. Analyses

The voltages were collected every 10 min with a precision multimeter using a data acquisition system (Keithley 2700, USA). When measuring the polarization curve, the external resistance used ranged from 100 to 5000 Ω. Stable voltages were then recorded at several resistance values. The current density and the power density were calculated according to the projected anodic surface area. The current (*I*) was calculated according to Ohm's Law: *U* = *IR*, where *U* is the voltage and *R* is the external resistance. Power (*P*) was calculated according to *P* = *IU*. Internal resistance was calculated via the polarization slope method [29]. Cyclic voltammetry (CV) was performed using a potentiostat (CHI660D, Shanghai Chen Hua Instrument Co. Ltd) in a three-electrode system. The working electrode was the cathode, while the anode and Ag/AgCl were used as the counter and reference electrodes, respectively. The potentials were shifted from −600 to 600 mV at a scan rate of 20 mV s^−1^ while measuring the current response.

The electrode surface morphologies were studied by scanning electron microscopy with coupled energy-dispersive spectroscopy (SEM–EDS; JSM-5900, Japan). The bacteria attached to the cathode were immobilized based on previous report [30]. The elemental composition and the oxidation states of the Cr species on the cathode surface were analysed by X-ray photo electron spectroscopy (XPS) with a PHI 5000 VersaProbe Spectrometer (UlVAC-PHI, Japan). The residual soluble Cr(VI) of the samples was filtered and total soluble chromium performed using the standard colorimetric method [31].

## 3. Results and discussion

### 3.1. Formation and characterization of the RVC-CNT biocathode

**Figure 1.**
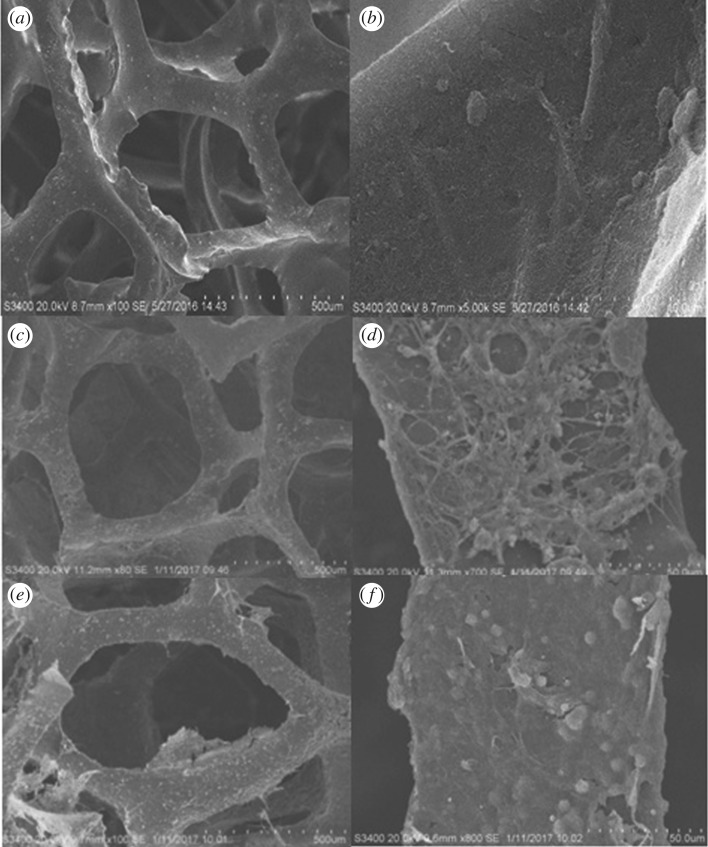
SEM of (*a*,*b*) RVC-CNT; (*c*,*d*) RVC biocathode; (*e*,*f*) RVC-CNT biocathode before the Cr(VI)-removal experiment in low and high scale, respectively.

The SEM images illustrate that the RVC is a continuous three-dimensional scaffold, with a pore size of approximately 400 µm (figure 1*a*). The macroporous structure allows biofilm formation and is beneficial to substrate mass transfer. More importantly, it provides a larger specific surface area for CNT coating. The CNT was successfully deposited onto the RVC surface without damaging its original macroporous structure (figure 1*b*) through the EPD technique. The RVC biocathode surface has many microorganisms that formed a thin loose biofilm layer (figure 1*c*,*d*). The results showed that the *ex situ* acclimation method increased the microbial densities in the biocathode, compared to the common acclimation method in the cathode chamber of the Cr(VI)-removal MFC [7]. Furthermore, more microorganisms were attached on the RVC-CNT biocathode and formed a continuous, dense biofilm (figure 1*e*,*f*). This may be due to the presence of CNT, which was conducive to microbial adhesion, thus forming a three-dimensional CNT/biofilm network structure.

### 3.2. Electricity generation

**Figure 2.**
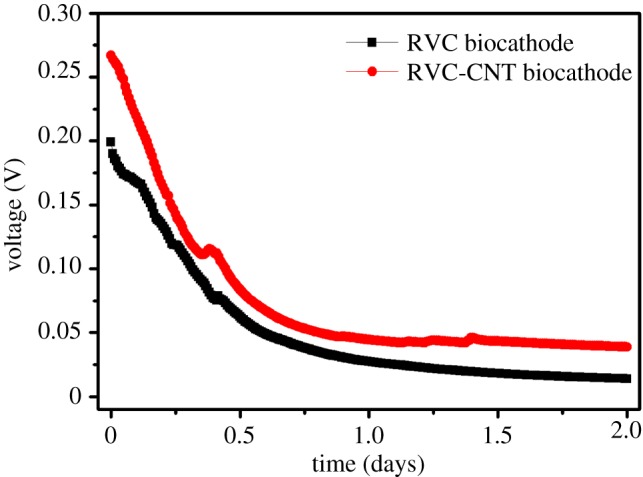
Voltage out of the Cr(VI)-removal MFCs with different biocathodes. Data represent the average value of duplicate experiments.

For all MFCs, the MFC voltage continuously decreased when 20 mg l^−1^ of Cr(VI) concentration was added into the reactor (figure 2), and a similar phenomenon was observed in the typical Cr(VI)-removal MFC [7,9,10]. The initial voltage of the MFC with an RVC-CNT biocathode was 267 ± 8 mV, which was higher than that of the MFC with an RVC biocathode (199 ± 3 mV) (figure 2). Subsequently, the voltage of all the MFCs declined quickly in 15 h and then became stable. At the end of the experiment, the voltage of the MFC with an RVC-CNT biocathode was 39 ± 3 mV, whereas that of the MFC with an RVC biocathode was only 14 ± 2 mV.

**Figure 3.**
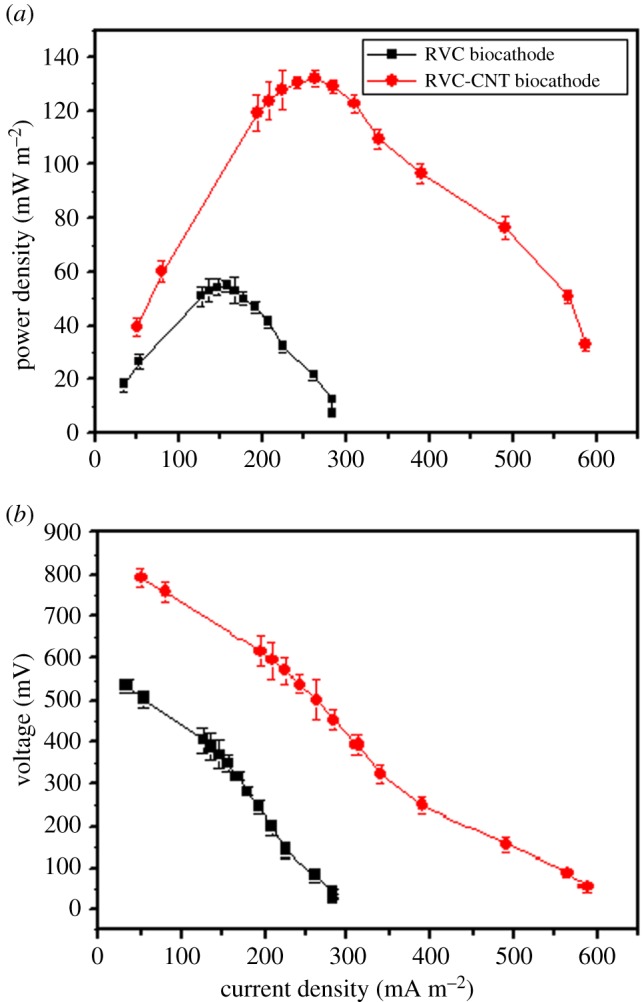
(*a*) Power density curves and (*b*) polarization curves of the Cr(VI)-removal MFCs with different cathodes.

Polarization curves were obtained from the Cr(VI)-removal MFCs when the voltage was maximal (figure 3*a*). The MFC with an RVC-CNT biocathode generated the higher maximum power density (*P*_max_) (132.1 ± 2.8 mW m^−2^) than the MFC with an RVC biocathode (55.0 ± 4.9 mW m^−2^). The MFC with the RVC-CNT biocathode was 2.4 times that of an MFC with the RVC biocathode. The internal resistance of MFC was estimated from the slope of the plot of the voltage opposite current (figure 3*b*). A high internal resistance (664 ± 8 Ω) was observed in the MFC with an RVC cathode, whereas the internal resistance of MFC with an RVC-CNT biocathode (446 ± 10 Ω) was relatively low, thus resulting in high output power in the MFC with an RVC-CNT biocathode.

### 3.3. Cr(VI) reduction

**Figure 4.**
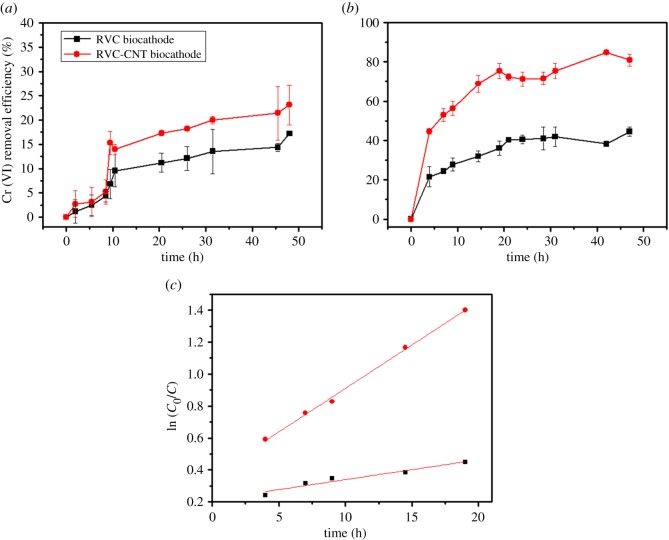
Time course of Cr(VI) concentration in the cathode chamber of MFCs (*a*) the open-circuit condition and (*b*) the close-circuit condition (*c*) ln (*C*_0_/*C*).

CNT possesses high specific surface areas and a porous structure, which makes it a good adsorbent for heavy metal removal [32,33]. Thus, the Cr(VI) removal in the MFCs with different cathodes was investigated under open-circuit (there was no current) and closed-circuit conditions to analyse the Cr(VI) removal effect (adsorption or bioelectrochemical reduction). Under open-circuit conditions (figure 4*a*), the Cr(VI) removal efficiency in the MFC with an RVC-CNT biocathode quickly reached 13.9 ± 3.3% within 10 h, the Cr(VI) removal efficiency in the MFC became slowed later, and the final Cr(VI) removal efficiency was 23.1 ± 4.0% at 48 h. On the contrary, the Cr(VI) removal efficiency in the MFC with an RVC biocathode reached 9.5 ± 3.3% at 10 h and 17.2 ± 0.4% at 48 h. The Cr(VI) removal was mainly due to the physical adsorption of the electrode under open-circuit conditions and the deposition of CNT on the electrode provided a large specific surface area, which is beneficial for the adsorption of more hexavalent chromium. Once the circuit was closed, the removal efficiency of Cr(VI) increased in all MFCs (figure 4*b*). The removal efficiency of Cr(VI) in the MFC with an RVC-CNT biocathode reached 80.9 ± 2.9% within 48 h, which was higher than that with an RVC biocathode (44.5 ± 2.3%). The removal efficiency of Cr(VI) with the closed-circuit RVC biocathode and RVC-CNT biocathode were enhanced 2.6 and 3.5 times, respectively, compared to those achieved under the open-circuit control, indicating that the bioelectrochemical process stimulated the removal of Cr(VI).

The removal of Cr(VI) occurred mainly in 20 h. The Cr(VI) removal efficiency increased quickly and followed a pseudo first-order model: ln (*C*_0_/*C*) = *k* × *t*, where *C*_0_ is the initial Cr(VI) concentration, mg l^−1^; *C* is the dissolved Cr(VI) at time *t*, mg l^−1^; *t* represents the time, h; and *k* is the pseudo first-order rate constant, h^−1^. The results of the linear curve fit over time are demonstrated in figure 4*c*. The MFC with an RVC-CNT biocathode had a high *k* value (0.054 h^−1^, *R*^2^ = 0.99), and the MFC with an RVC biocathode had a low value (*k*, 0.012 h^−1^, *R*^2^ = 0.92), suggesting that the RVC-CNT biocathode improved the removal of the Cr(VI) compared with the RVC biocathode. Furthermore, the Cr(VI) removal rate of 0.78 ± 0.04 mg l^−1^ h^−1^ was obtained for the MFC with an RVC-CNT biocathode, which was 2.1 times higher than that obtained for the MFC with an RVC biocathode.

Under the same *ex situ* acclimatization method, the MFC with graphite felt biocathode had a Cr(VI) reduction rate of 0.66 ± 0.01 mg l^−1^ h^−1^ at 20 mg l^−1^ Cr(VI) in a previous report [8], which was higher than that of the MFC with an unmodified RVC biocathode, but it was lower than that of the MFC with a RVC-CNT biocathode. Furthermore, the projected electrode surface area of graphite felt (25 cm^2^) in the previous reported Cr(VI)-removal MFC [8] was larger than that of the RVC-CNT (12.5 cm^2^) in our experiment. Therefore, based on the projected electrode surface area, the Cr(VI) reduction rate of the RVC-CNT biocathode was significantly improved compared to that of the graphite felt. The results strongly suggest that the three-dimensional structure of the CNT-modified RVC could enhance the bioelectrocatalytic activity for Cr(VI) reduction.

### 3.4. Possible mechanism of Cr(VI) reduction by RVC-CNT biocathode

**Figure 5.**
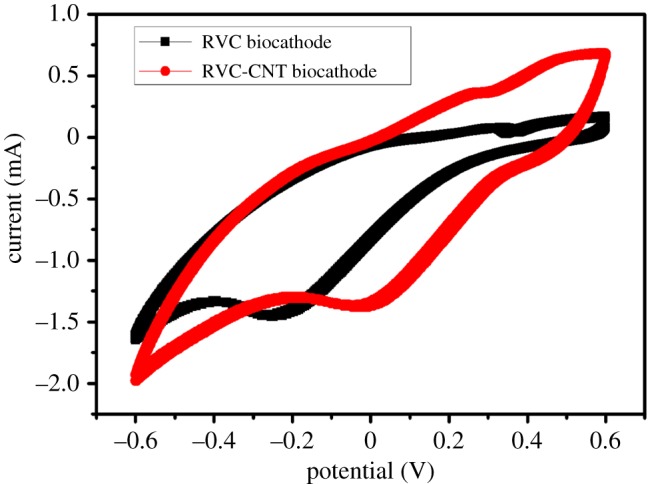
Cyclic voltammogram curves of the RVC biocathode and the RVC-CNT biocathode.

Our previous report indicated that Cr(VI) removal in biocathode MFCs through an *ex situ* acclimation method was mediated by the cathodic microbial community [8]. To test the electrochemical activity of the cathodes, the catalytic activities of the biofilms on different biocathodes were studied by CV (figure 5) at the end of the experiment. The cathodic currents (negative) caused by the reduction in the Cr ions on the electrode were followed by an anodic current (positive) caused by the re-oxidation of the reduced Cr during the reverse scan. The deposition of Cr on the electrode surface may facilitate hydrogen generation, caused by the reduction in H^+^ to H_2_ gas, thus causing the appearance of a reductive peak. An obvious reduction peak, at approximately −0.25 V (versus Ag/AgCl), was observed for the RVC biocathode, while a reduction peak at about −0.02 V was observed for the RVC-CNT biocathode. The positions of the oxidation–reduction peaks reveal the redox potential of the electron transfer components of the bacteria [32]. The reduction peak position of the RVC-CNT biocathode was higher than that of the RVC biocathode. Furthermore, the MFC with an RVC-CNT biocathode visualized maximum current in both the forward scan (0.65 mA) and the reverse scan (−1.97 mA), and a lower current output (0.08 mA, −1.64 mA) was observed in the MFC with RVC biocathode. The high reduction peak position and current response indicate that CNT deposition could improve the electron transfer capability of the RVC biocathode.

**Figure 6.**
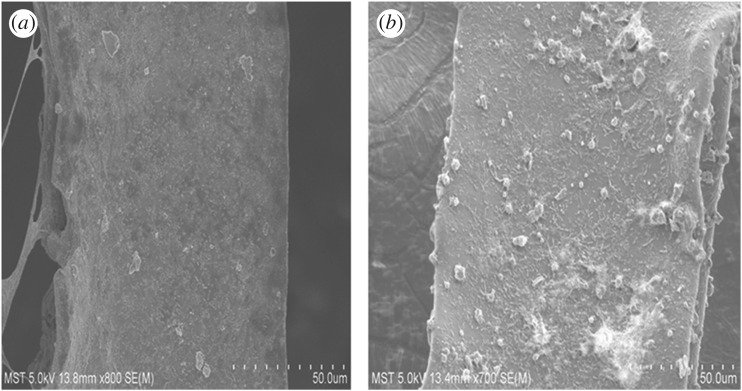
SEM images of (*a*) RVC biocathode and (*b*) RVC-CNT biocathode at the end of experiment.

**Figure 7.**
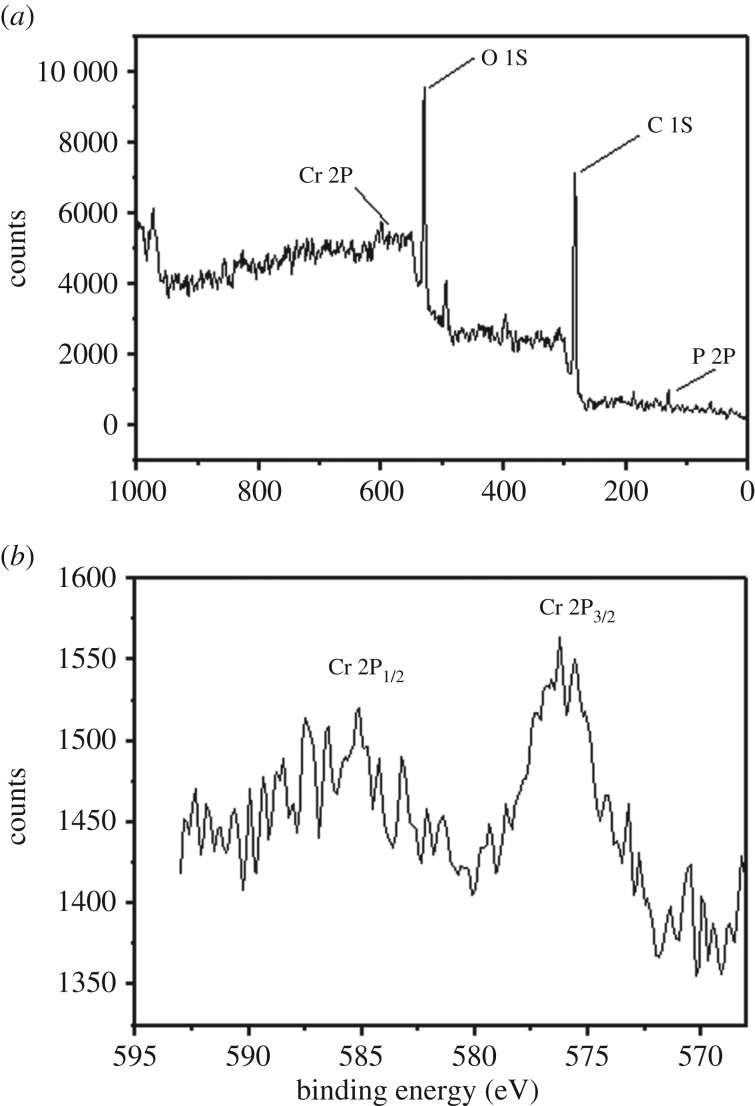
EDS of (*a*) RVC biocathode and (*b*) RVC-CNT biocathode at the end of experiment.

At the end of the experiment, the cathodes were re-analysed by SEM (figure 6). Noticeable precipitated particles covered the surface of all cathodes. The precipitated particles on the RVC electrode were relatively sparse, while more precipitated particles were observed and were denser in some places on the RVC-CNT electrode. The composition of these particles was further analysed by EDS (table 1). The signal value for Cr in the RVC-CNT biocathode was 13.41%, which was higher than that of the RVC biocathode (0.83%). The EDS results indicated that more chromium deposited on the RVC-CNT biocathode at the end of the experiment. The elemental composition of the RVC-CNT biocathode was further verified by XPS. The elements of C, O, Cr and P were present in the RVC-CNT biocathode (figure 7*a*). Cr 2p_1/2_ and Cr 2p_3/2_ lines were observed at 576.9 and 586.7 eV, respectively. In the Cr 2p spectrum (figure 7*b*) of the RVC-CNT biocathode, the results implied that Cr(III) was deposited upon the electrode surface and formed Cr(III) phosphate [33]. The large quantity of Cr(III) deposits on the RVC-CNT biocathode was attributed to its high reduction rate of Cr(VI), which may adopt an adsorption–reduction mechanism. The CNT deposited onto the RVC surface can decrease the Cr(VI) concentration by physical adsorption. Adsorbing Cr(VI) onto the surface of the electrode and reducing the mass transfer resistance of the oxidized hexavalent chromium to the reducing electrode allow more substrates to participate in the reaction. More importantly, the three-dimensional CNT/biofilm network structure enhanced the electron transfer rate from the biofilm to the Cr(VI), and thus the bacteria on the cathode may transfer electrons from the cathode to the Cr(VI). These bacteria can produce mediators and nanowires that transfer electrons between the bacteria and the electrode indirectly or directly [34,35]. The Cr(VI) reduction rate can be enhanced via the bacterial biofilms used as biocatalyst (biocathodes), thus the increasing electron transfer rate due to the RVC-CNT biocathode could significantly improve the reduction rate of Cr(VI) in the MFC.

**Table 1.** EDS spectra of different biocathodes at the end of the experiment.

	RVC biocathode		RVC-CNT biocathode	
element	Wt%	At%	Wt%	At%
C K	50.52	67.15	47.29	62.41
O K	24.65	24.60	25.53	26.03
Al K	0.18	0.11		
Si K	0.71	0.41		
S K	0.98	0.49	7.10	3.61
K K	5.96	2.43		
Cr K	0.83	0.25	13.41	4.21
Fe K	14.90	4.26	6.67	1.95
Zn K	1.26	0.31		

In the Cr(VI)-removal MFC, graphite felt, graphite fibre, graphite plate and graphite granules [7,12–14] were reported as capable biocathode materials. Furthermore, graphite felt can also be modified by NaX zeolite [36] or graphene [10], to improve the affinity for microorganisms and Cr(VI) ions or the electrical conductivity of the electrodes as well as the electron transfer rate. The combination of good materials and optimum surface electrode modification is a promising strategy to fabricate composite electrodes for microbial electrocatalysis [11]. Compared to the graphite felt, RVC has attracted significant attention for its usage as three-dimensional electrode in MFC for electricity generation due to its exceptionally high void volume, high surface area and rigid structure [18,37]. It allows bacteria to enter the inner structure of the three-dimensional electrode to form an electrocatalytically active biofilm. However, the internal resistance of RVC itself was relatively large (664 ± 8 Ω). Therefore, RVC-CNT biocathode was fabricated by the EPD method, to decrease internal resistance (446 ± 10 Ω) in this study. Actually, the CNT-based RVC biocathodes have been studied in many bioelectrochemical systems, such as microbial electrosynthesis (MES) system [38]. In their MES system, CO_2_ was reduced to acetate through the NanoWeb-RVC biocathode. The biofilm area and the mass transfer efficiency of the MES system were maximized because of the high surface area to volume ratio of the macroporous RVC [38]. Here, a RVC-CNT biocathode was adopted in a Cr(VI) removal system for the first time. Unlike the biofilm formed autotrophically on the NanoWeb-RVC in the MES system, a dense and firm biofilm was heterotrophically formed in our anodic chamber first and transferred to cathodic chamber to catalyse the Cr(VI) reduction reaction. A dense CNT structure can improve the electrical conductivity of RVC, as well as increase the communication with bacteria and Cr(VI). Thus, the enhanced reduction rate of Cr(VI) in MFC has been achieved. In addition, the EPD process was relatively simple and can produce a uniform CNT film, which was suitable for the large-scale electrode modification in Cr(VI)-removal MFC. Further work should focus on the preparation of the RVC-CNT electrode, such as the choice of RVC with different pore sizes and the optimization of the EPD process.

## 4. Conclusion

In this study, a simple and effective method for fabricating three-dimensional biocathodes for Cr(VI)-removal MFC through the EPD of CNT on the RVC was demonstrated. The RVC-CNT biocathode significantly improved the electricity generation (132.1 ± 2.8 mW m^−2^) and Cr(VI) reduction rate (0.78 ± 0.04 mg l^−1^ h), which were 2.4 and 2.1 times higher than those on the unmodified RVC. These improvements might be due to the numerous adsorption sites and the high electron transfer rate in the CNT/biofilm network structure.

## Data accessibility

All data are included within the article.
